# Supplementary figures and images for: In silico Analysis of Genetic Diversity of Human Hepatitis B Virus in Southeast Asia, Australia and New Zealand
Source: Viruses. 2020 Apr 9;12(4):427. doi: 10.3390/v12040427 (PMC7232418; doi:10.3390/v12040427)

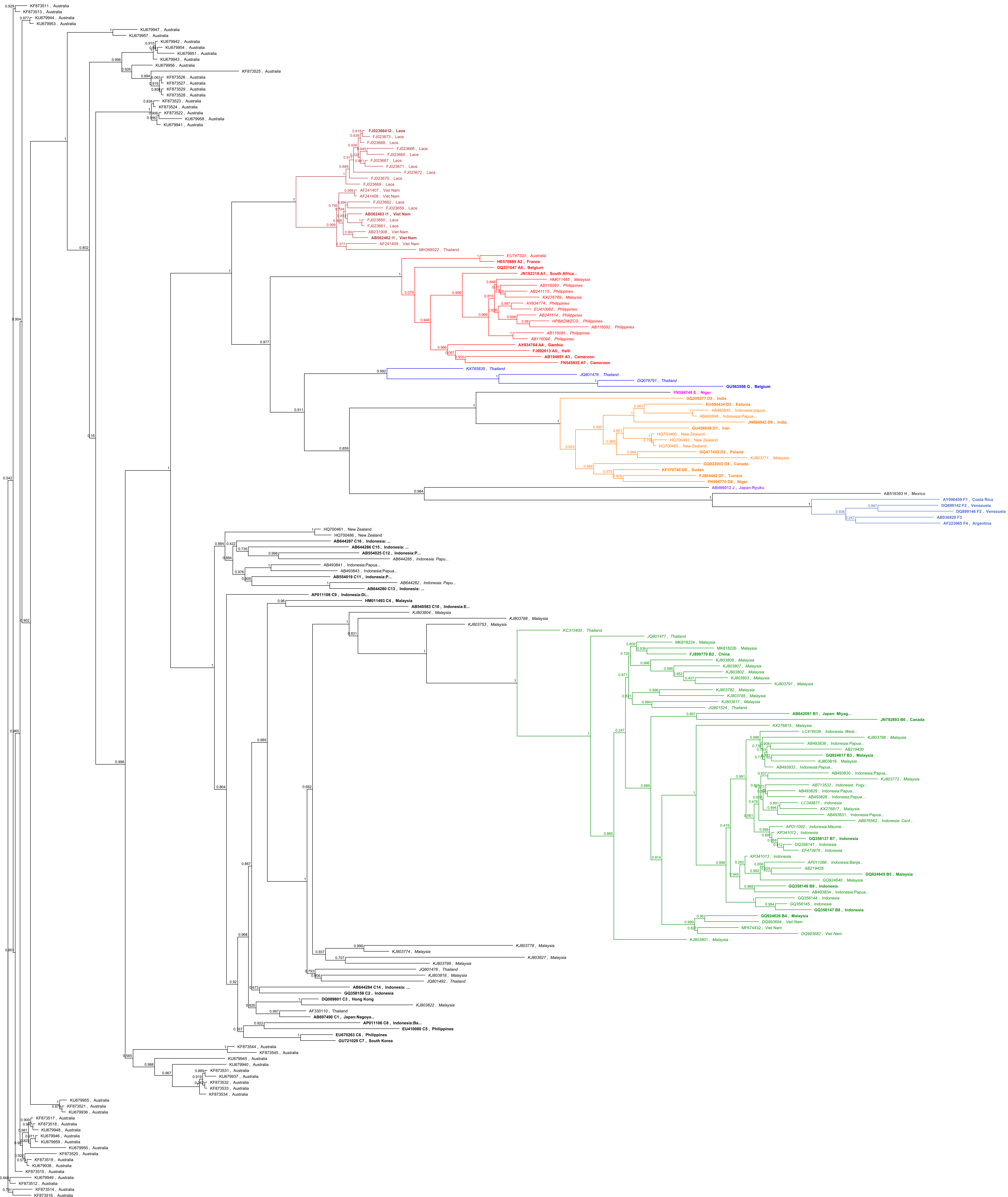

Supplement: Supplementary file 1 [file viruses-12-00427-s001.zip › Figure S1- FastTree tree of ambiguous genotypes and recombinants.pdf]

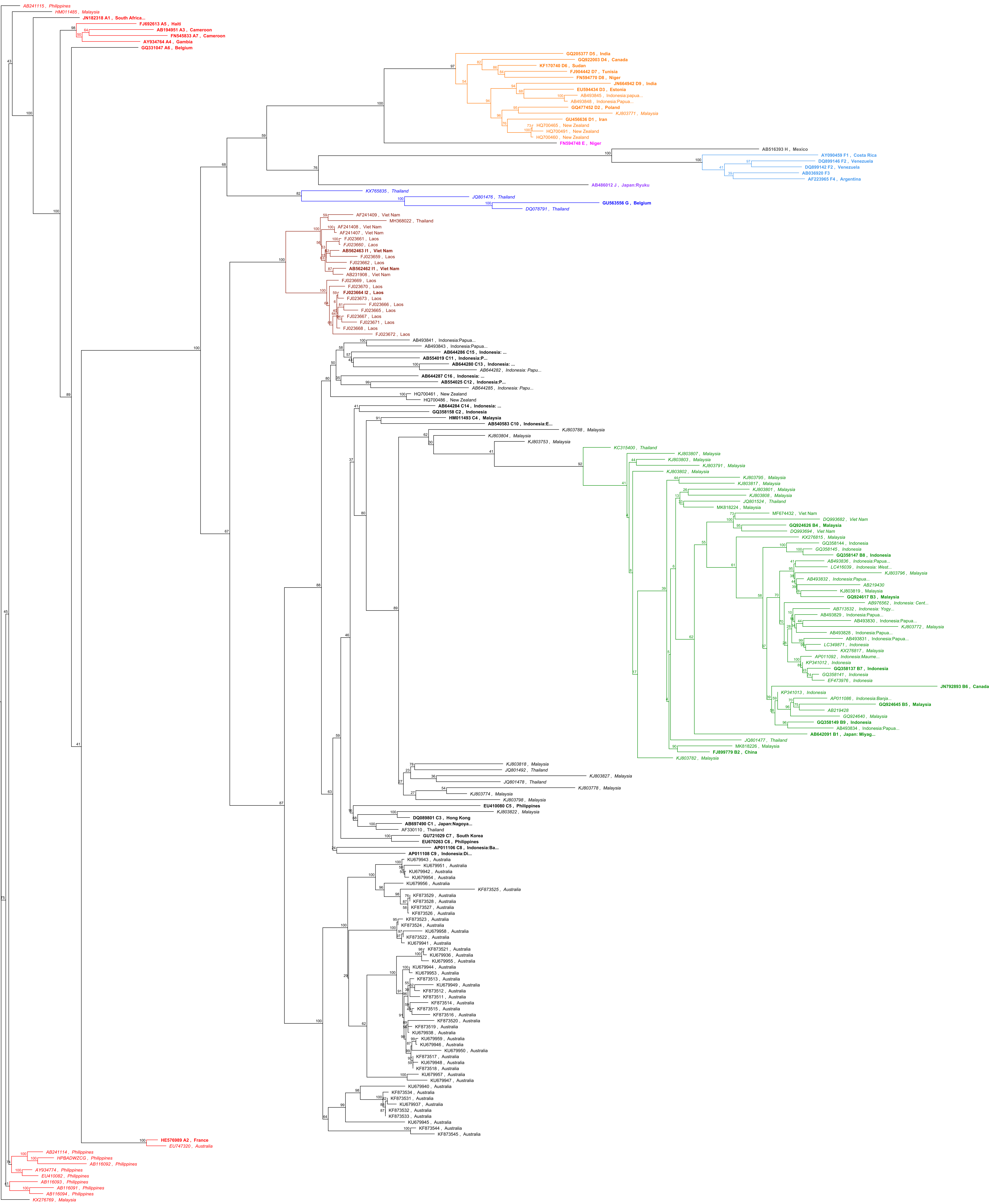

Supplement: Supplementary file 1 [file viruses-12-00427-s001.zip › Figure S2- RAxML tree of ambiguous genotypes and recombinants.pdf]

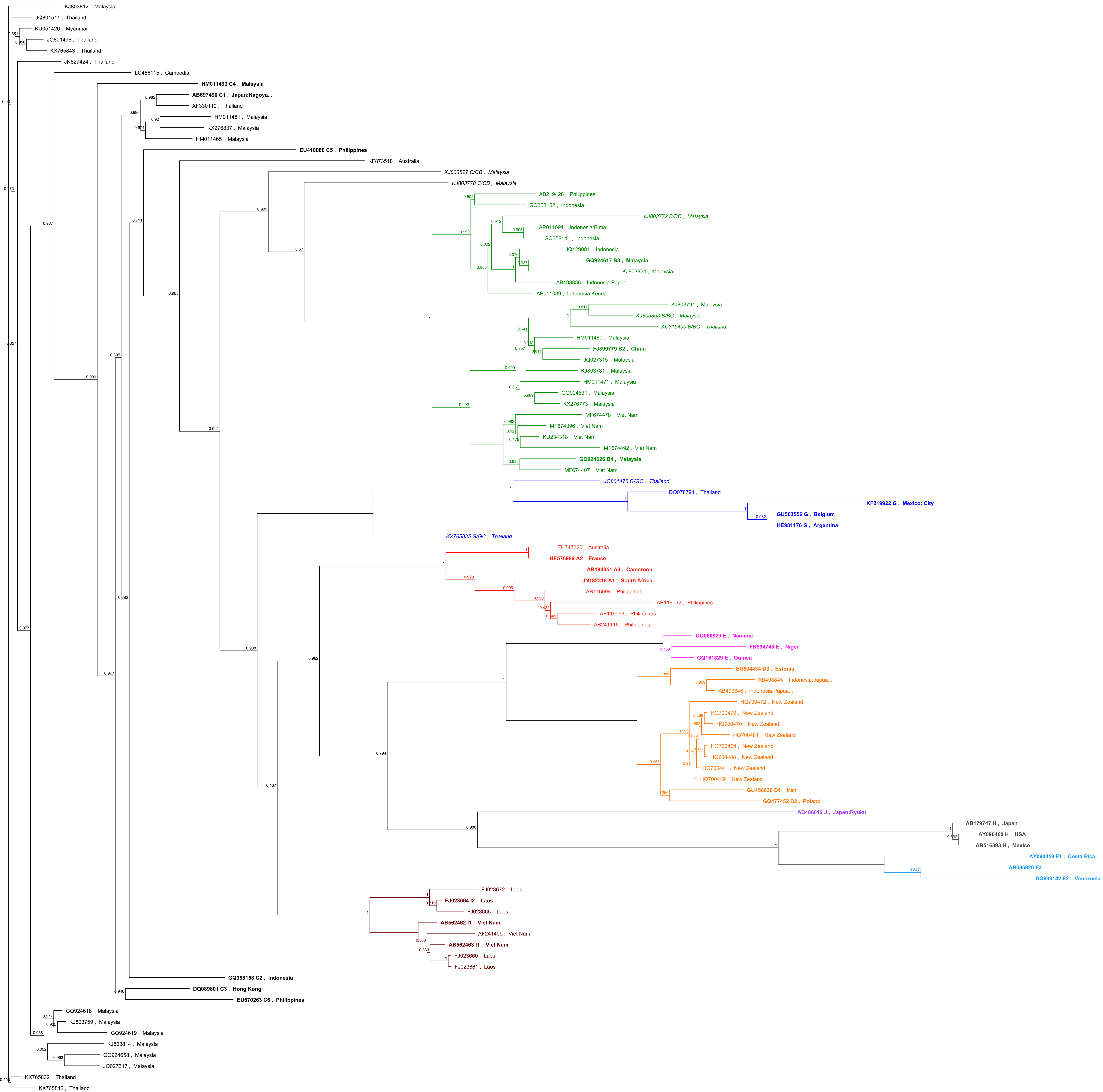

Supplement: Supplementary file 1 [file viruses-12-00427-s001.zip › Figure S3- FastTree tree of presentative population.pdf]

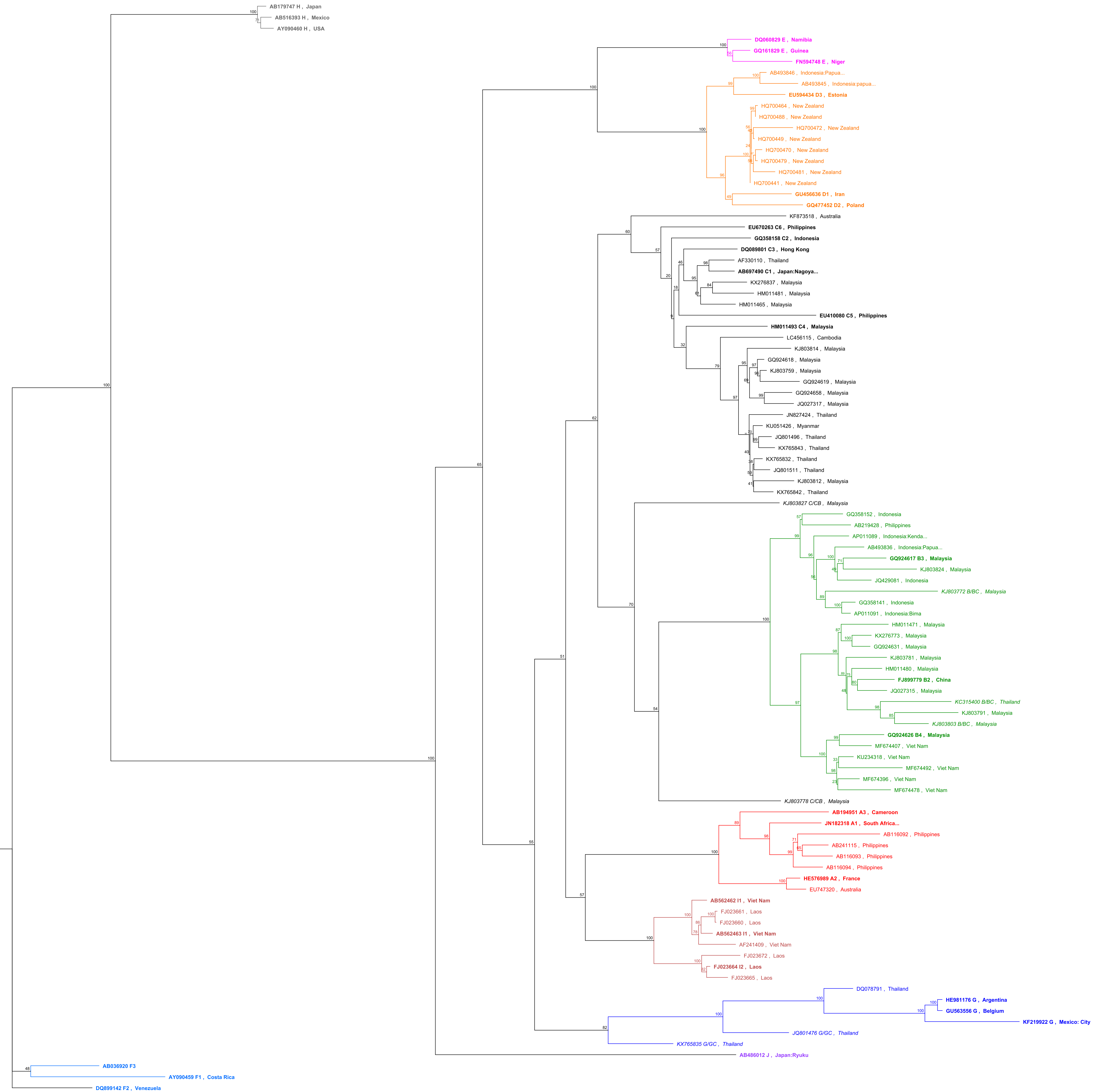

Supplement: Supplementary file 1 [file viruses-12-00427-s001.zip › Figure S4- RAxML tree of presentative population.pdf]

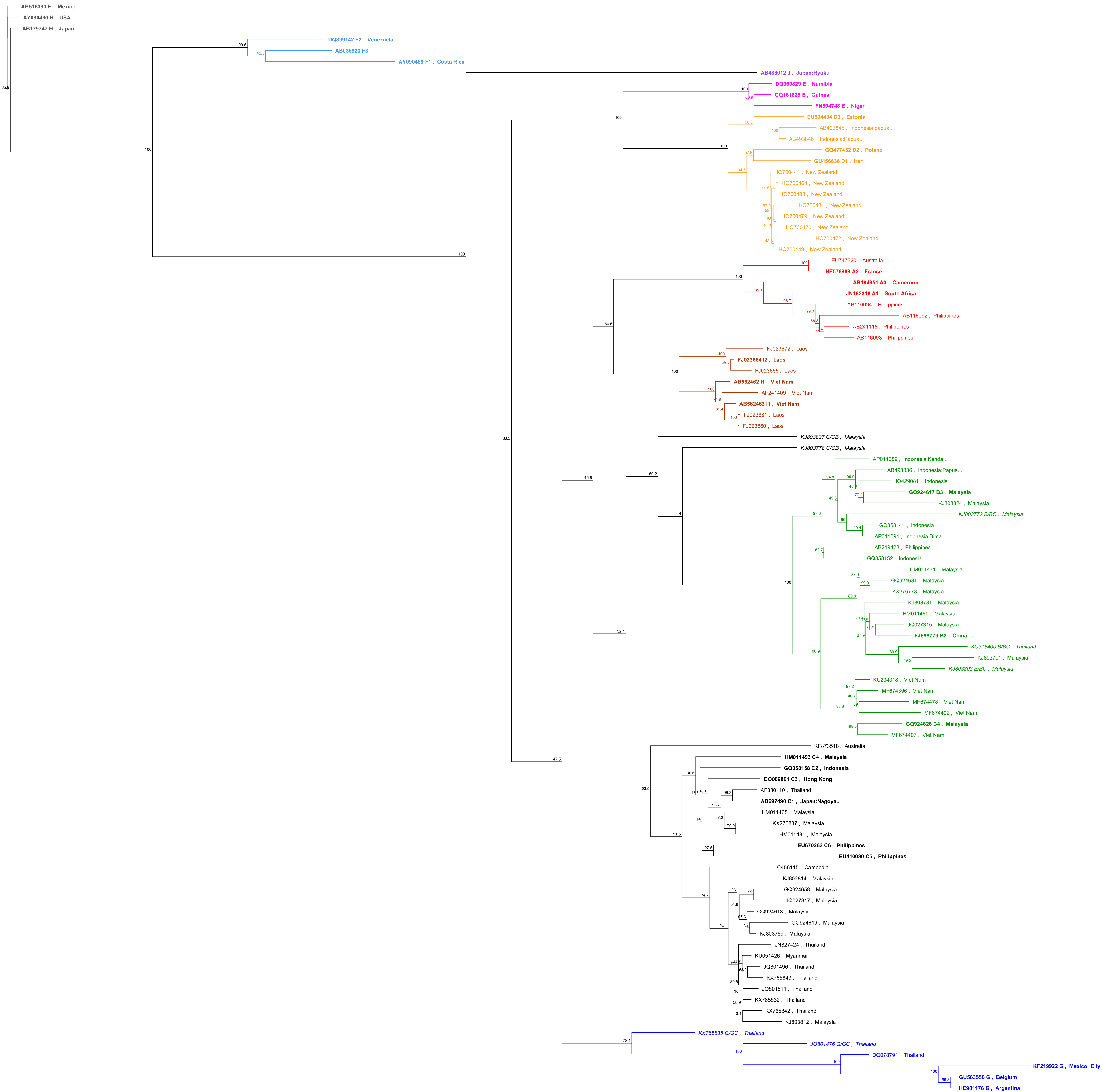

Supplement: Supplementary file 1 [file viruses-12-00427-s001.zip › Figure S5- PHYML tree of presentative population.pdf]
