# Supplementary material for: In silico Analysis of Genetic Diversity of Human Hepatitis B Virus in Southeast Asia, Australia and New Zealand
Source: Viruses. 2020 Apr 9;12(4):427. doi: 10.3390/v12040427 (PMC7232418; doi:10.3390/v12040427)
Supplement: Supplementary file 1 [file viruses-12-00427-s001.zip › viruses MDPI - supplementary materials.doc]

Article

Genetic diversity of human hepatitis B virus in Southeast Asia, Australia and New Zealand estimated *in silico*

Ngoc Minh Hien Phan ^1, 2*^, Helen Faddy ^1, 2, 3^, Robert Flower ^1, 2^, Kirsten Spann ^1, 2^ and Eileen Roulis ^1,2^

^1^	Faculty of Health, School of Biomedical Sciences, Queensland University of Technology, 149 Victoria Park Rd, Kelvin Grove, Queensland, Australia, 4059; [kirsten.spann@qut.edu.au](mailto:kirsten.spann@qut.edu.au)

^2^	Department of Research and Development, Brisbane Processing Centre, Australian Red Cross Lifeblood, 44 Musk Avenue, Kelvin Grove, Queensland, Australia, 4059; [hphan@redcrossblood.org.au](mailto:hphan@redcrossblood.org.au); [rFlower@redcrossblood.org.au](mailto:rFlower@redcrossblood.org.au); [eroulis@redcross.blood.org.au](mailto:eroulis@redcross.blood.org.au)

^3^	University of Sunshine Coast; [hfaddy@usc.edu.au](mailto:hfaddy@usc.edu.au)

*****	Correspondence: [hphan@redcrossblood.org.au](mailto:hphan@redcrossblood.org.au); Tel: +61 738389382

Received: date; Accepted: date; Published: date

Supplementary materials

**Supplementary file 1.** Summarised results of 930 human HBV genomes from Southeast Asia, Australia and New Zealand (file attached)

**Supplementary figure 1.** FastTree maximum likelihood tree built from sequences of ambiguous genotypes and potential recombinants identified by RDP4. Genotypes are colour-coded. Reference sequences with their genotypes were in bold. 65 sequences with potential recombination events identified by five programs of RDP4 were in italics.

**Supplementary figure 2.** RAxML maximum likelihood tree build from sequences of ambiguous genotypes and potential recombinants identified by RDP4. Genotypes are colour-coded. Reference sequences with their genotypes were in bold. Sequences with potential recombination events identified by five programs of RDP4 were in italics.

**Supplementary figure 3.** FastTree maximum likelihood tree built from representative population. Genotypes are colour-coded. Reference sequences with their genotypes were in bold. Seven identified recombinants were in italics.

**Supplementary figure 4.** RAxML maximum likelihood tree built from representative population. Genotypes are colour-coded. Reference sequences with their genotypes were in bold. Seven identified recombinants were in italics.

**Supplementary figure 5.** PHYML maximum likelihood tree built from representative population. Genotypes are colour-coded. Reference sequences with their genotypes were in bold. Seven finally identified recombinants were in italics.

**Supplementary Table 1.** Reference sequences used in this study

| Genotype | References used to recheck ambiguous genotypes /recombinants | References for phylogenetic analysis of representative population | References for recombination analysis with RDP4 |
| --- | --- | --- | --- |
| A | A1: JN182318.1  A2: HE576989.1  A3: AB194951.1  A4: AY934764.1  A5: FJ692613.1  A6: GQ331047.1  A7: FN545833.1 | A1: JN182318.1  A2: HE576989.1  A3: AB194951.1 | A1: KU736920.1, KM519454.1  A2: HE576989.1, HE974364.1  A3: HM363613.1, HM363612.1  A4: KM606737.1  A5: FJ692554.1  A6: GQ331048.1, GQ331046.1  A7: FN545833 |
| B | B1: AB642091.1  B2: FJ899779.1  B3: GQ924617.1  B4: GQ924626.1  B5: GQ924645.1  B6: JN792893.1  B7: GQ358137.1  B8: GQ358147.1  B9: GQ358149.1 | B2: FJ899779.1  B3: GQ924617.1  B4: GQ924626.1 | B1: AB900112.1, KP341007.1  B2: GQ377612.1, JX869998  B3: KP341009.1  B4: KP341010.1  B5: GQ924645.1 |
| C | C1: AB697490.1  C2: GQ358158 .1  C3: DQ089801 .1  C4: HM011493.1  C5: EU410080.1  C6: EU670263.1  C7: GU721029.1  C8: AP011106.1  C9: AP011108.1  C10: AB540583.1  C11: AB554019.1  C12: AB554025.1  C13: AB644280.1  C14: AB644284.1  C15: AB644286.1  C16: AB644287.1 | C1: AB697490.1  C2: GQ358158 .1  C3: DQ089801 .1  C4: HM011493.1  C5: EU410080.1  C6: EU670263.1 | C1: KP017266.1, GQ377605.1, GU357843.1  C2: HQ622095.1, FJ787487.1  C3: KU695741.1  C4: GQ377635.1, GQ377630.1  C5: KM999992.1  C6: GQ358155.1  C7: LC416040.1  C8: MG826131.1  C9: AP011108  C10: KY629637.1  C11: AB554019  C12: AB554025  C13: AB644280  C14: AB644284  C15: AB644286  C16: AB644287 |
| D | D1: GU456636.1  D2: GQ477452.1  D3: EU594434.1  D4: GQ922003.1  D5: GQ205377.1  D6: KF170740.1  D7: FJ904442.1  D8: FN594770.1  D9: JN664942.1 | D1: GU456636.1  D2: GQ477452.1  D3: EU594434.1 | D1: GU357846.1, KY382414.1  D2: MH724241.1, KR905423.1  D3: KC012652.1, KP090178.1  D4: KJ470897.1, GQ922003.1  D5: KP322603.2  D6: KF170740.1  D7: KU736923.1, KM606741.1 |
| E | FN594748.1 | FN594748.1, DQ060829.1, GQ161829.1 | EU23922.1 |
| F | F1: AY090459.1  F2: DQ899146.1, DQ899142.1  F3: AB036920.1  F4: AF223965.1 | F1: AY090459.1  F2: DQ899142.1  F3: AB036920.1 | F1: KP995116.1, FJ589065.1  F2: KT896494.1, KP995115.1  F3: KP995126.1, FJ589067.1  F4: KY382411.1, HE981181.1 |
| G | GU563556.1 | GU563556.1  KF219922.1  HE981176.1 | HE981176.1, KF219922.1 |
| H | AB516393.1 | AB516393.1, AY090460.1, AB179747.1 |  |
| I | I1: AB562463.1, AB562462.1  I2: FJ023664.1 | I1: AB562463.1, AB562462.1  I2: FJ023664.1 | I1: GU357844.1  I2: FJ023670.1 |
| J | AB486012.1 | AB486012.1 | AB486012.1 |

**Supplementary Table 2.** Features of finally identified potential recombinants ^1^

| Sequence ID | Overlapped breakpoint region (start – end) ^2^ | Same major parents  (Yes/No) ^3^ | Highest similarity score (compared to corresponding references) |
| --- | --- | --- | --- |
| KJ803827 C/CB | 831 - 1632 | Yes, by 4 methods | 93.406% (compared to AB697490\|C1)  93.250% (compared to GQ358149\|B9) |
| KJ803778 C/CB | 982 - 1395 | Yes, by 4 methods | 93.375% (compared to DQ089801\|C3)  93.064% (compared to GQ358149\|B9) |
| KJ803772 B/BC | 1824 - 2001 | No | 95.894% (compared to GQ358137\|B7)  91.944% (compared to AB697490\|C1) |
| KJ803803 B/BC | 3202 – 8 | Yes, by 2 methods | 96.641% (compared to FJ899779\|B2)  92.597% (compared to AB697490\|C1) |
| KC315400 B/BC | 1078 - 1579 | Yes, by 5 methods | 96.128% (compared to FJ899779\|B2)  93.359% (compared to AB697490\|C1) |
| JQ801476 G/GC | 3184 – 3197 | Yes, by 3 methods | 92.006% (compared to AB697490\|C1)  91.841% (compared to GU563556\|G) |
| KX765835 G/GC | 2135 – 2505 | Yes, by 4 methods | 93.468% (compared to DQ089801\|C3)  91.756% (compared to GU563556\|G) |

^1^ The recombinants were raised by HBV genotyping and further confirmed by HBV recombination detection and phylogenetic analyses

^2^ Positions were relative to AB033554. The breakpoint positions given by the five programs were considered altogether to see whether and how long they were overlapping to each other.

^3^ For each of the detected recombinants with overlapping breakpoint positions, we compared its major parents recognised by the automatic recombination detection methods in RDP4 to determine whether they were repeatedly identified by more than one program.

**Supplementary File 1.** Summarised results of 930 human HBV genomes from Southeast Asia, Australia and New Zealand

The results were described in a separate excel document.
